# Real-World Effectiveness of Natalizumab in Korean Patients With Multiple Sclerosis

**DOI:** 10.3389/fneur.2021.714941

**Published:** 2021-07-08

**Authors:** Ki Hoon Kim, Su-Hyun Kim, Na Young Park, Jae-Won Hyun, Ho Jin Kim

**Affiliations:** Department of Neurology, Research Institute and Hospital of National Cancer Center, Goyang, South Korea

**Keywords:** disease modifying therapy, multiple sclerosis, natalizumab, progressive multifocal leukoencephalopathy, annual relapse rate

## Abstract

**Background and Purpose:** Natalizumab is a highly efficacious disease-modifying therapy for relapsing-remitting multiple sclerosis (MS). Data on the efficacy and safety profile of natalizumab in Asian patients with MS are limited. This study assessed the efficacy and safety of natalizumab in Korean patients with MS in a real-world setting.

**Methods:** This study enrolled consecutive Korean patients with active relapsing-remitting MS who were treated with natalizumab for at least 6 months between 2015 and 2021. To evaluate the therapeutic outcome of natalizumab, we used the Expanded Disability Status Scale (EDSS) scores and brain magnetic resonance imaging; adverse events were assessed at regular intervals. No evidence of disease activity (NEDA) was defined as no clinical relapse, no worsening of EDSS score, and no radiological activities.

**Results:** Fourteen subjects with MS were included in the study. The mean age at initiation of natalizumab therapy was 32 years. All patients were positive for anti-John Cunningham virus antibodies before natalizumab administration. The mean annual relapse rate was markedly reduced from 2.7 ± 3.2 before natalizumab therapy to 0.1 ± 0.4 during natalizumab therapy (*p* = 0.001). Disability was either improved or stabilized after natalizumab treatment in 13 patients (93%). During the 1st year and 2 years after initiating natalizumab, NEDA-3 was achieved in 11/12 (92%) and 9/11 (82%) patients, respectively. No progressive multifocal leukoencephalopathy or other serious adverse events leading to the discontinuation of natalizumab were observed.

**Conclusions:** Natalizumab therapy showed high efficacy in treating Korean patients with active MS, without unexpected safety problems.

## Introduction

Multiple sclerosis (MS) is a chronic, immune-mediated, inflammatory demyelinating disease of the central nervous system (CNS) that carries a high risk of disability (1). Natalizumab, a recombinant humanized monoclonal antibody against the α4-integrin cell adhesion molecule, is a highly efficacious disease-modifying therapy (DMT) for active MS (2, 3). After two pivotal phase 3 clinical studies (AFFIRM and SENTINEL), natalizumab was approved for relapsing-remitting MS (RRMS) in 2004 by the US Food and Drug Administration and in 2015 by the Korea Food and Drug Administration (4–6). Observational studies demonstrated the long-term effectiveness and well-established safety profile of natalizumab (7, 8). However, the predominant concern with the use of natalizumab is its association with the development of progressive multifocal leukoencephalopathy (PML), a rare serious opportunistic infection caused by the John Cunningham virus (JCV), which can cause severe disability or even death. Prior use of immunosuppressants, longer treatment duration (especially >2 years), and the presence of JCV antibody with higher JCV antibody index values are known to increase the risk of developing PML in natalizumab-treated patients (9). Thus, clinicians can stratify patients according to the risk of PML based on the JCV antibody status. Recent studies have reported a higher seroprevalence of anti-JCV antibodies and a higher proportion of patients with an elevated index (>1.5) among Asian patients with MS compared with that of Western cohorts (10–12). Considering these results, neurologists in Asia might be more concerned about the risk of PML and hence be reluctant to prescribe natalizumab for patients with MS (11).

To date, data on the efficacy and safety of natalizumab in Asian patients have been limited to a 24-week phase 2 clinical trial of natalizumab and a study of post-marketing surveillance in Japan (13, 14).

In this study, we aimed to evaluate the real-world effectiveness and safety of natalizumab treatment in Korean patients with MS.

## Methods

### Subjects

This study enrolled consecutive Korean patients with RRMS who were treated with natalizumab for at least 6 months at the National Cancer Center (NCC) in Korea between November 2015 and March 2021, and we retrospectively analyzed their medical records and magnetic resonance imaging (MRI) (15). Of enrolled 14 patients, two patients were treatment-naïve and 12 patients were previously treated with other DMTs (interferon-beta, glatiramer acetate, dimethyl fumarate [DMF], teriflunomide, fingolimod, and alemtuzumab) but showed breakthrough disease activity [relapses or new/enlarging T2 lesions or gadolinium (Gad)-enhancing T1 lesions on MRI]. Intravenous natalizumab 300 mg was administered monthly for 1 h, and infusion-associated reactions (IARs) were monitored for up to 1 h after infusion. Prior to initiating natalizumab therapy, a serological test for anti-JCV antibody was performed. After 24 monthly administrations, natalizumab therapy was discontinued and replaced with natalizumab-exit medication in all patients due to the risk of PML except for one patient who was unable to use natalizumab-exit medication due to an unexpected pregnancy. This study was approved by the Institutional Review Board of the NCC (NCC2014-0146), and informed consent was obtained from all the patients.

### Efficiency and Safety Measures

Baseline expanded disability status scale (EDSS) score was measured right before the first administration of natalizumab, and baseline brain MRI scans were obtained within 6 months before natalizumab administration. EDSS was assessed by an independent, but unblinded neurologist. During natalizumab therapy, EDSS and brain MRI scans were performed regularly every 6 months. Clinical relapses were defined as new or worsening neurological symptoms lasting at least 24 h, confirmed by neurological examination, and unrelated to fever or infection (4, 5).

Clinical efficacy was evaluated by the annual relapse rate (ARR) and the change in the EDSS score. Disability worsening was defined as an increase of 1 or more points from baseline if the baseline EDSS score was >0 or an increase of 1.5 or more points if the baseline EDSS score was 0 (4, 5). Disability improvement was defined as a decrease of 1 or more points from baseline among patients with baseline EDSS scores ≥2.0 (7). Serial brain MRI scans were used to evaluate radiological activities. New or enlarging T2 lesions and newly detected Gad-enhancing T1 lesions were compared with baseline MRI scans to evaluate radiological disease activities. In addition, no evidence of disease activity (NEDA-3)—defined as no clinical relapse, no worsening of disability as measured by the EDSS scores, and no radiological activity—during 1 and 2 years of treatment was also used to determine the efficacy of natalizumab (16). Clinical relapses and radiological activities were primarily determined by the treating neurologists (HJK, SHK), and confirmed by one of other neurologists (SHK, JWH, KHK). For the evaluation of NEDA-3, patients who showed an evidence of disease activities during natalizumab treatment were included in the analysis, even though the treatment period did not meet the index. After 2 years of natalizumab therapy, rebound was defined as at least one clinical relapse plus rebound activity on MRI (>5 Gad-enhancing lesions and a higher number of new enlarging and/or Gad-enhancing lesions than before initiation of natalizumab treatment during washout or for 6 months following new DMT initiation after cessation of natalizumab) (17).

For safety outcomes, clinical information regarding adverse events, infection events, and IARs was prospectively collected. Adverse events and IARs were assessed every month at each infusion. IARs were defined as any event developing within 2 h after the initiation of natalizumab infusion (4, 5). Regular laboratory tests including complete blood count, liver function test, serum creatinine, and urinalysis, were performed at least every 3 months.

### Statistical Analysis

Data are reported as number (percentage), mean (range), mean ± standard deviation, or median (interquartile range) for the EDSS score. The Wilcoxon signed-rank test was used to compare pre-natalizumab ARR and ARR during natalizumab treatment. Differences were regarded as significant with a *p-*value <0.05.

## Results

### Patients

Baseline demographics and clinical features are displayed in Table 1. The mean age of the patients at natalizumab start was 32 years (range, 13–41), and the mean duration from disease onset to natalizumab initiation was 59 months (range, 3–135). The male-to-female ratio was 1:1. Two patients with high disease activity used natalizumab as the first DMT, and 12 (86%) patients used one or more DMTs prior to natalizumab administration, comprising of interferon-beta, teriflunomide, DMF, glatiramer acetate, fingolimod, and alemtuzumab in 8, 6, 5, 2, 2, and 1 patient, respectively. The median number of previous DMTs before initiating natalizumab therapy was 2 (range, 0–5). The median baseline EDSS score was 2.5 [interquartile range (IQR), 1.0–3.0]. All patients were positive for anti-JCV antibodies before natalizumab administration. Thirteen (93%) patients had experienced a clinical attack 1 year before the initiation of natalizumab, and Gad-enhancing lesions were detected on the baseline brain MRI in all patients.

**Table 1:** Baseline demographics, clinical characteristics, and NTZ treatment outcome.

**Baseline demographics and clinical characteristics**	**Patients (*n* = 14)**
Age at onset, years	27 (13–41)
Age at NTZ start, years	32 (19–52)
Sex, male	7 (50)
Onset to NTZ start, months	59 (3–135)
Number of previous DMTs before NTZ	2 [1–2]
0	2 (14)
1	4 (29)
2	6 (43)
3	0 (0)
4	1 (7)
5	1 (7)
Baseline EDSS	2.5 [1.0–3.0]
Presence of anti-JCV antibody before NTZ	14 (100)
Presence of enhancing lesion on baseline brain MRI	14 (100)
**NTZ treatment duration and therapeutic outcome**
NTZ treatment duration
≥24 times	9 (64)
<24 times	5 (36)
Ongoing NTZ treatment	3 (21)
Discontinuation of NTZ treatment	2 (14)
Number of clinical attacks during NTZ treatment	1
EDSS progression during NTZ treatment	1 (7)
Disability improvement during NTZ treatment	3 (21)
NEDA-3 after initiating NTZ
During 1st year of follow-up	11/12 (92)
During 2 years of follow-up	9/11 (82)
ARR
Pre-NTZ	2.7 ± 3.2
Pre-NTZ, (recent, 1 year)	1.8 ± 1.4
Pre-NTZ, treatment naïve	3.4 ± 3.7
Pre-NTZ, under DMTs	2.3 ± 2.6
During NTZ treatment	0.1 ± 0.4

*Data are presented as mean ± standard deviation, mean (range), median [interquartile range], or N (%) value*.

*NTZ, natalizumab; DMTs, disease-modifying therapies; EDSS, expanded disability status scale; NEDA, no evidence of disease activity; ARR, annual relapse rate*.

### Treatment Duration of Natalizumab and Therapeutic Outcome

**Figure 1:**
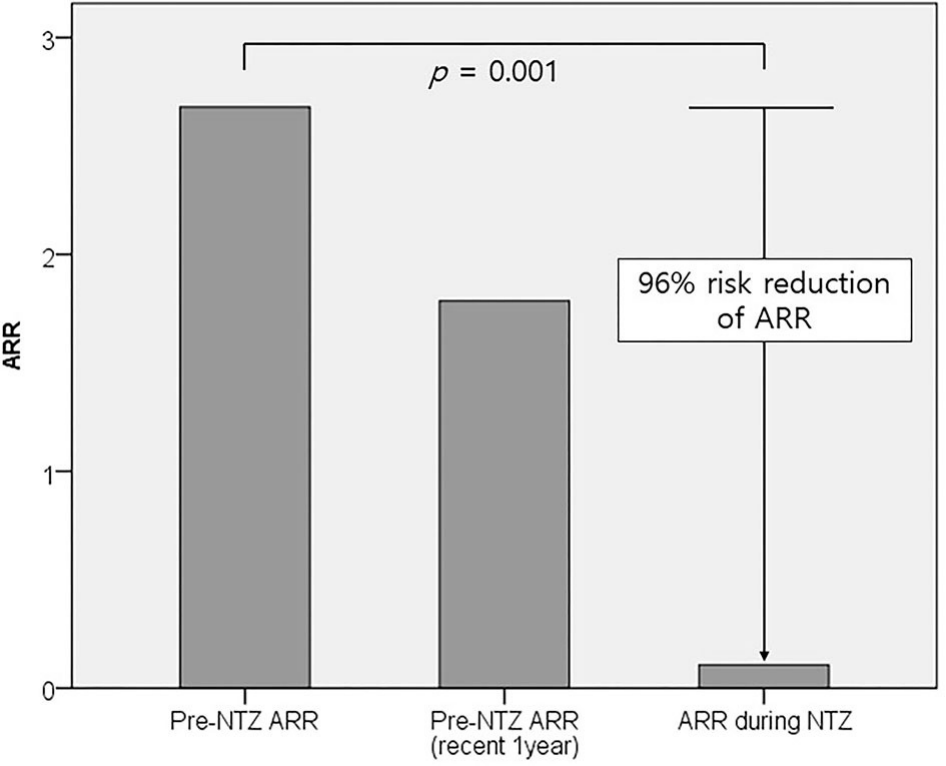
Comparison of mean ARRs before and during natalizumab therapy. The mean ARR significantly decreased from 2.7 ± 3.2 before natalizumab administration to 0.1 ± 0.4 during natalizumab therapy (*p* = 0.001). ARR, annual relapse rate; NTZ, natalizumab.

The treatment duration and therapeutic outcomes of natalizumab are summarized in Table 1. Nine patients completed 24 monthly administrations of natalizumab: two of them received more than 24 (one 25 times and the other 29 times). Three patients were still receiving natalizumab therapy and had received 20, 9, and 6 monthly administrations, respectively. The other 2 (14%) stopped natalizumab before 24 administrations; one patient stopped the treatment after 11 administrations due to logistic issues, and the other patient after 18 administrations due to lack of efficacy based on EDSS progression. This patient showed disability progression indicated by worsening of EDSS scores (baseline 3.0 > 3.5 > 4.0 > 5.5) without clinical relapse or new MRI lesions on three follow-up scans over 18 months, which suggested progression independent of relapse activity. Of a total of 286 monthly administrations in 14 patients, only one clinical relapse occurred 2 weeks after the first administration of natalizumab. The mean ARR was markedly reduced from 2.7 ± 3.2 before natalizumab administration to 0.1 ± 0.4 during natalizumab therapy, representing a 96% risk reduction (*p* = 0.001, Figure 1). On natalizumab therapy, 6-month confirmed disability worsening was observed in 1 (7%) patient, and 3 (21%) patients experienced disability improvement as measured by the change in the EDSS scores. In addition, new or enlarging T2 lesions and Gad-enhancing T1 lesions were not observed on follow-up brain MRI scans in any of the patients during natalizumab therapy. During 1 and 2 years after initiating natalizumab, NEDA-3 was achieved in 11/12 (92%) and 9/11 (82%) patients, respectively. Two patients who were treated with natalizumab as the first DMT achieved NEDA-3 during the 2 years of natalizumab therapy.

### Safety

The IARs and adverse events associated with natalizumab therapy are listed in Table 2. Two (14%) patients experienced IARs, including facial flushing and headache. These IARs occurred after the first administration of natalizumab. The degree of IAR was mild and spontaneously resolved without symptomatic medication. Anaphylactic events, such as hypotension or desaturation, were not observed.

**Table 2:** Safety profile during NTZ therapy.

	**Patients (*n* = 14)**
**Infusion-associated reactions**
Facial flushing and redness	1 (7)
Headache	1 (7)
**Infection events**
Upper respiratory tract infection	2 (14)
Nasopharyngitis (common cold)	2 (14)
Urinary tract infection	1 (7)
Tonsillitis	1 (7)
Boil and pus at groin area	1 (7)
Otitis media	1 (7)
**Other adverse events**
Diarrhea	2 (14)
Liver enzyme elevation	1 (7)
Joint pain (ankle)	1 (7)
Alopecia	1 (7)
Chronic headache	1 (7)
Restless leg syndrome	1 (7)

*Data are N (%) value*.

*NTZ, natalizumab*.

None of the patients showed unexpected serious adverse events (SAEs), such as malignancy or PML during the median follow-up duration of 36.6 months (range, 6.6–66.8) from the initiation of natalizumab therapy. Infections on natalizumab treatment occurred eight times in 7 (50%) patients. Upper respiratory tract infection and nasopharyngitis were observed in two patients. Urinary tract infection, tonsillitis, boil in the groin area, and otitis media each developed in one patient. All infection events were successfully treated without complications and did not lead to natalizumab discontinuation.

Intermittent diarrhea, which was self-limiting without medication, developed in two patients. Joint pain at the ankle, alopecia, and headache were each developed in one patient. One of the patients developed an unpleasant feeling on the leg and a strong urge to move her leg at night after six administrations of natalizumab. Under the diagnosis of restless leg syndrome, her symptoms were controlled by a dopamine agonist and disappeared after 3 months. One patient showed an increased liver enzyme level (2–3 times the normal upper limit) after the 10th infusion, which spontaneously stabilized after 5 months.

### Clinical Course After 24 Monthly Infusions of Natalizumab

**Figure 2:**
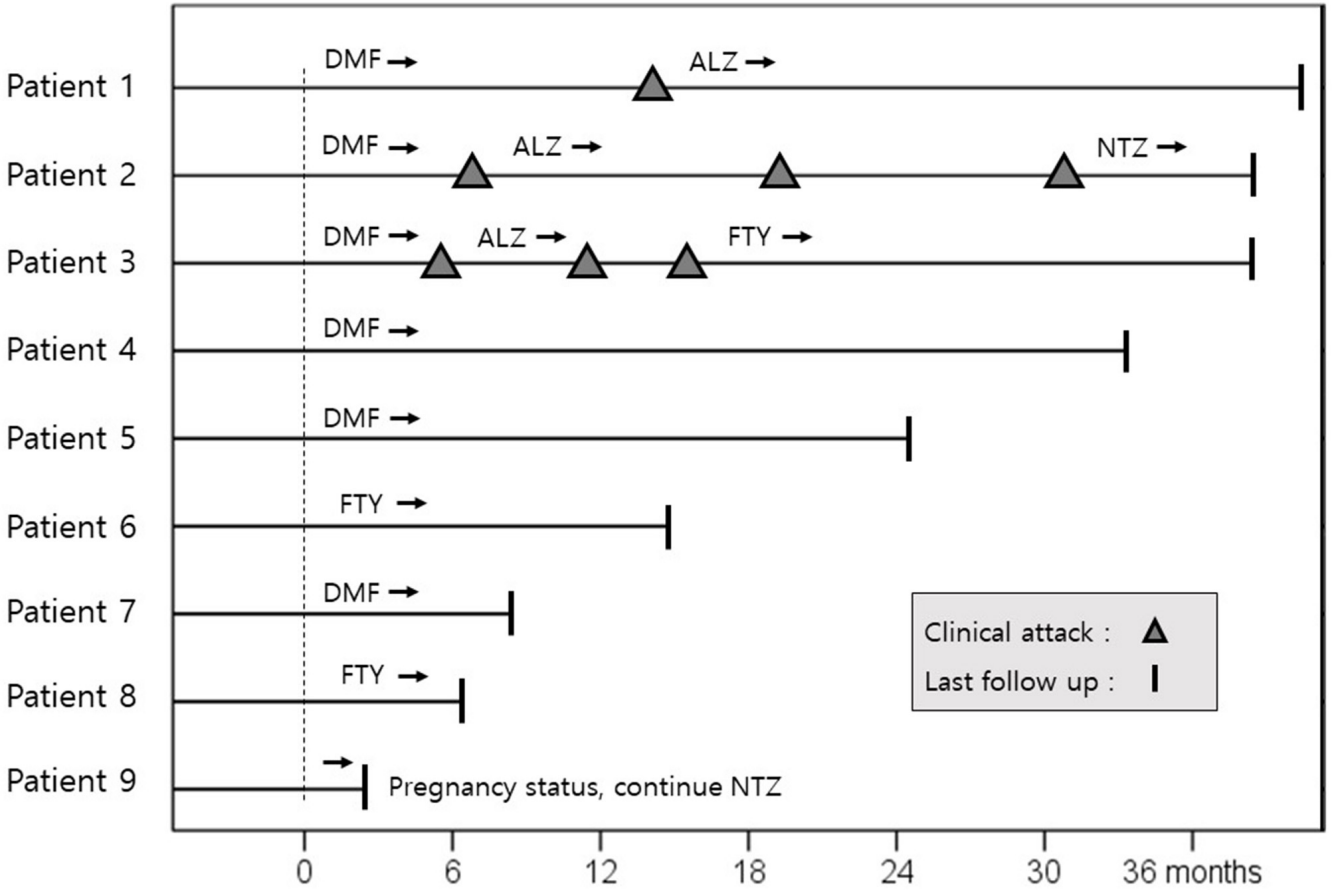
Clinical course of nine patients after successful treatment of NTZ. The dotted line is the time point at which 24 or more monthly infusions of natalizumab had been administered. Three of six patients, who selected dimethyl fumarate as a natalizumab exit strategy, experienced clinical relapses after 2 years of successful NTZ treatment. Dimethyl fumarate and fingolimod were administered with a washout period of 4 and 6 weeks, respectively. ALZ, alemtuzumab; DMF, dimethyl fumarate; NTZ, natalizumab; FTY, fingolimod.

Nine patients completed 24 or more administrations of natalizumab (Figure 2). These nine patients achieved NEDA-3 during natalizumab therapy. After successful natalizumab treatment, DMF and fingolimod were chosen as natalizumab-exit medications with a washout duration of 4 and 6 weeks for six and two patients, respectively. One patient continued to receive natalizumab treatment because commonly used oral DMTs could not be given as natalizumab-exit medications due to unexpected pregnancy. Of the six patients who chose DMF as a natalizumab-exit medication, three experienced relapses 4, 5, and 12 months after the initiation of DMF. These three patients were subsequently treated with alemtuzumab, and one patient was stable without further relapse for 20 months. However, two of them experienced four further clinical relapses and showed high disease activity on brain MRI under alemtuzumab therapy. In one patient, myelitis occurred 10 months after first cycle alemtuzumab, and myelitis developed again 10 months after second cycle alemtuzumab with a new Gad-enhancing T1 lesion on brain MRI. The second patient experienced two myelitis relapses 5 and 8 months after 1st cycle alemtuzumab. Of the two patients who had relapses despite alemtuzumab treatment, one patient switched to fingolimod and was stable without further clinical relapse for 22 months, while the other patient recently requested to restart natalizumab treatment despite the high risk of PML, as most of the currently available DMTs had failed to control his disease. Two patients who chose fingolimod as a natalizumab-exit medication were stable for 15 and 6 months, respectively. In addition, two patients (patients 4 and 7 in Figure 2), who used natalizumab as the first DMT, switched natalizumab to DMF and showed no clinical relapse for 33 and 7 months after natalizumab treatment, respectively. Rebound was seen in one patient who chose DMF as a natalizumab-exit medication 5 months after the initiation of DMF.

## Discussion

In real-world studies conducted in Western populations, pre-natalizumab ARRs decreased by 73–100% during natalizumab treatment, and 33–63% of patients achieved NEDA for 1.3 to 2.0 years of the mean follow-up duration (18, 19). In terms of safety, SAEs were reported in 0–6% of patients, including PML, ranging in incidence from 0.03 to 0.4% (19). The proportion of patients showing IARs has been reported to range from 0 to 12%. In a clinical phase 2 trial for Japanese patients, the therapeutic outcome was not different from that of Western patients; 77% of patients achieved NEDA during 24 weeks of natalizumab treatment, and the mean ARR for the 30 months of natalizumab treatment was 0.13 (13, 14). Death or PML cases have not been reported in Japanese patients.

In this study, 82% of patients achieved NEDA-3 and 93% showed stabilized or improved disability during the 2-years of natalizumab therapy. Only one patient experienced a minor clinical relapse, but recovered completely after 1 week. A 96% risk reduction in mean ARR was observed during natalizumab therapy. No radiological activities were found on the MRI scans regularly performed every 6 months in all patients. No PML or other SAEs leading to the discontinuation of natalizumab were observed. Although it is difficult to compare clinical outcomes directly due to the small number of enrolled patients, the efficacy and safety of natalizumab in the Korean population seem comparable with those reported in previous real-world Western studies and phase 2 clinical trials conducted in Japanese patients.

A major obstacle regarding the use of natalizumab is the risk of PML. The incidence of natalizumab-related PML was reported to be 0.9%, and the overall incidence rate per 1,000 patient-years was 2.03 in a recent 10-year observational study (7). It is well-known that the probability of natalizumab-related PML rises steeply in patients with previous immunosuppressive therapy who have received the medication for more than 24 months and are seropositive for anti-JCV antibodies (20). Our patients were all seropositive for anti-JCV antibody before natalizumab therapy, and no sign or radiologic evidence of PML was found throughout the 2 years of natalizumab treatment. Given the higher seroprevalence of JCV antibody and its higher index in Asian patients, there might be some concerns as to whether Asian patients are more susceptible to natalizumab-related PML (10–12). This study is a real-world experience of natalizumab in the Asian population, and supports the current PML risk stratification for natalizumab-treated Asian patients, in whom 2 years of natalizumab treatment is unlikely to pose a risk of PML.

Despite the high efficacy of natalizumab, the risk of PML usually limits the duration of natalizumab therapy to 24 months. Discontinuation of natalizumab may restore disease activities to the state they were before natalizumab treatment; thus, the selection of an appropriate natalizumab-exit strategy is important (18). Published data on the use of pulsed corticosteroids, interferon-beta, and glatiramer acetate show that these agents cannot prevent rebound activity, and breakthrough disease activity occurs in a proportion of patients with MS treated with either DMF or teriflunomide (21–24). Notably, 50% of our patients who selected DMF as a natalizumab exit strategy experienced clinical relapses on DMF treatment. Fingolimod has been the most studied and preferentially used DMT after cessation of natalizumab, and it seems to pose a lower risk of relapse than interferon beta or glatiramer acetate (18, 25). In terms of “carryover PML,” which develops several months after the discontinuation of natalizumab and when starting a new DMT, fingolimod has an advantage over alemtuzumab and cladribine, which induce long-term immunosuppression followed by delayed immune reconstitution (26). If “carryover PML” is developed on fingolimod, stopping fingolimod shortly allows immune reconstitution and makes cytotoxic T cells traffic into the CNS to control JCV infection (26). In addition, to prevent rebound disease activity, most experts recommend starting fingolimod with a short washout period (within 6–8 weeks) (25, 27). An extended interval dosing (EID) can be an alternative approach to reduce the risk of PML, while not losing the efficacy. A retrospective study with 1974 natalizumab-treated patients showed that an EID up to 8 weeks did not reduce effectiveness of natalizumab therapy (28), and a subsequent study showed that EID was associated with a lower risk of PML than a standard interval dosing (SID) (29). An ongoing randomized prospective trial of EID vs. SID will provide a comprehensive evidence of EID.

Recent studies have reported that active control of disease activity with high-efficacy drugs in the early stage of MS offer sustained clinical benefits on delaying the disability progression and conversion to SPMS (30–32). Treatment strategy for patients with high disease activity is evolving into individualized therapeutic models that initiate high-efficacy DMT in the disease onset and early in the course of disease. This strategy implements an individualized benefit-risk assessment to balance the level of DMT efficacy with the risk of drug-related adverse events considering the disease activity or the risk of disease progression (33). The majority of patients in our study failed on their first and second-line treatments and showed high disease activity prior to natalizumab treatment and thus had a high risk of rapid progression of disability. Nonetheless, natalizumab treatment achieved the high rate of NEDA-3 over 2 years and no serious adverse events including PML occurred. Our results suggest natalizumab treatment in Asian patients with high disease activity for 2 years is possibly an effective and safe therapeutic option while not increasing the risk of PML.

This study has several limitations. First, this study was single-centered with a small sample size (*n* = 14), and hance, there's a possibility of selection bias. Second, the follow-up durations from the initiation of natalizumab (median 36.6 months) were not long enough to reflect the long-term safety of natalizumab. Third, we were unable to compare the anti-JCV antibody index before and after natalizumab treatment, because only the presence or absence of anti-JCV antibody was provided without the anti-JCV antibody index until 2017. In a previous Western study, the anti-JCV antibody index significantly increased during natalizumab treatment and following natalizumab discontinuation (34). In order to better stratify the potential risk of PML in Asian populations whose seroprevalence and index values of anti-JCV antibodies are very high, it is necessary to further investigate the temporal change of anti-JCV antibody index.

Despite the aforementioned shortcomings, the reassuring results of this study, which confirm the real-world effectiveness and safety profile of natalizumab in Korean patients with MS, may prompt clinicians to overcome their reluctance to prescribe this medication. Further investigations on long-term disability outcomes, patient-reported outcomes, quality of life, and changes in anti-JCV antibody index in natalizumab-treated Korean patients are warranted.

## Data Availability Statement

The raw data supporting the conclusions of this article will be made available by the authors, without undue reservation.

## Ethics Statement

The studies involving human participants were reviewed and approved by the Institutional Review Board of the NCC (NCC2014-0146). The patients/participants provided their written informed consent to participate in this study.

## Author Contributions

KK, S-HK, and HK: conceptualization. KK, NP, J-WH, S-HK, and HK: resources. S-HK and HK: supervision. KK: visualization and writing—original draft. All authors writing—review and editing.

## Conflict of Interest

J-WH has received a grant from the National Research Foundation of Korea. HK received a grant from the National Research Foundation of Korea and research support from Aprilbio and Eisai; received consultancy/speaker fees from Alexion, Aprilbio, Biogen, Celltrion, Daewoong, Eisai, GC Pharma, HanAll BioPharma, MDimune, Merck Serono, Novartis, Roche, Sanofi Genzyme, Teva-Handok, UCB, and Viela Bio; is a co-editor for the Multiple Sclerosis Journal and an associated editor for the Journal of Clinical Neurology. The remaining authors declare that the research was conducted in the absence of any commercial or financial relationships that could be construed as a potential conflict of interest.

## References

[1] Filippi M Bar-Or A Piehl F Preziosa P Solari A Vukusic S. Multiple sclerosis. Nat Rev Dis Primers. (2018) 4:43. 10.1038/s41572-018-0050-3 30410033

[2] Steinman L. Blocking adhesion molecules as therapy for multiple sclerosis: natalizumab. Nat Rev Drug Discov. (2005) 4:510–8. 10.1038/nrd1752 15931259

[3] Delbue S Comar M Ferrante P. Natalizumab treatment of multiple sclerosis: new insights. Immunotherapy. (2017) 9:157–71. 10.2217/imt-2016-0113 28004598

[4] Polman CH O'Connor PW Havrdova E Hutchinson M Kappos L Miller DH. A randomized, placebo-controlled trial of natalizumab for relapsing multiple sclerosis. N Engl J Med. (2006) 354:899–910. 10.1056/NEJMoa044397 16510744

[5] Rudick RA Stuart WH Calabresi PA Confavreux C Galetta SL Radue EW. Natalizumab plus interferon beta-1a for relapsing multiple sclerosis. N Engl J Med. (2006) 354:911–23. 10.1056/NEJMoa044396 16510745

[6] Kim W Kim HJ. Monoclonal antibody therapies for multiple sclerosis and neuromyelitis optica spectrum disorder. J Clin Neurol. (2020) 16:355–68. 10.3988/jcn.2020.16.3.355 32657055 PMC7354979

[7] Butzkueven H Kappos L Wiendl H Trojano M Spelman T Chang I. Long-term safety and effectiveness of natalizumab treatment in clinical practice: 10 years of real-world data from the tysabri observational program (TOP). J Neurol Neurosurg Psychiatry. (2020) 91:660–8. 10.1136/jnnp-2019-322326 32234967 PMC7279201

[8] Guger M Enzinger C Leutmezer F Di Pauli F Kraus J Kalcher S. Long-term outcome and predictors of long-term disease activity in natalizumab-treated patients with multiple sclerosis: real life data from the austrian MS treatment registry. J Neurol. (2021) 1–8. 10.1007/s00415-021-10559-w. [Epub ahead of print]. 33890167 PMC8505366

[9] Bloomgren G Richman S Hotermans C Subramanyam M Goelz S Natarajan A. Risk of natalizumab-associated progressive multifocal leukoencephalopathy. N Engl J Med. (2012) 366:1870–80. 10.1056/NEJMoa1107829 22591293

[10] Aoyama S Mori M Uzawa A Uchida T Masuda H Ohtani R. Serum anti-JCV antibody indexes in Japanese patients with multiple sclerosis: elevations along with fingolimod treatment duration. J Neurol. (2018) 265:1145–50. 10.1007/s00415-018-8813-z 29532286

[11] Kim SH Kim Y Jung JY Park NY Jang H Hyun JW. High seroprevalence and index of anti-john-cunningham virus antibodies in korean patients with multiple sclerosis. J Clin Neurol. (2019) 15:454–60. 10.3988/jcn.2019.15.4.454 31591832 PMC6785463

[12] Lau A Qiu W Kermode A Au C Ng A Wong A. High prevalence and indexes of anti-john cunningham virus antibodies in a cohort of chinese patients with multiple sclerosis. Mult Scler J Exp Transl Clin. (2018) 4:2055217318788699. 10.1177/2055217318788699 30038791 PMC6050819

[13] Saida T Kira JI Kishida S Yamamura T Ohtsuka N Dong Q. Natalizumab for achieving relapse-free, T1 gadolinium-enhancing-lesion-free, and T2 lesion-free status in Japanese multiple sclerosis patients: a phase 2 trial subanalysis. Neurol Ther. (2017) 6:153–9. 10.1007/s40120-016-0062-4 28078634 PMC5447555

[14] Saida T Kira JI Kishida S Yamamura T Ohtsuka N Ling Y. Safety and efficacy of natalizumab in japanese patients with relapsing-remitting multiple sclerosis: open-label extension study of a phase 2 trial. Neurol Ther. (2017) 6:39–55. 10.1007/s40120-016-0059-z 27921221 PMC5447552

[15] Polman CH Reingold SC Banwell B Clanet M Cohen JA Filippi M. Diagnostic criteria for multiple sclerosis: 2010 revisions to the mcdonald criteria. Ann Neurol. (2011) 69:292–302. 10.1002/ana.22366 21387374 PMC3084507

[16] Havrdova E Galetta S Hutchinson M Stefoski D Bates D Polman CH. Effect of natalizumab on clinical and radiological disease activity in multiple sclerosis: a retrospective analysis of the natalizumab safety and efficacy in relapsing-remitting multiple sclerosis (AFFIRM) study. Lancet Neurol. (2009) 8:254–60. 10.1016/S1474-4422(09)70021-3 19201654

[17] Fuentes-Rumí L Hernández-Clares R Carreón-Guarnizo E Valero-López G Iniesta-Martinez F Cabrera-Maqueda JM. Prevention of rebound effect after natalizumab withdrawal in multiple sclerosis. study of two high-dose methylprednisolone schedules. Mult Scler Relat Disord. (2020) 44:102311. 10.1016/j.msard.2020.102311 32593958

[18] Sellner J Rommer PS. A review of the evidence for a natalizumab exit strategy for patients with multiple sclerosis. Autoimmun Rev. (2019) 18:255–61. 10.1016/j.autrev.2018.09.012 30639651

[19] van Pesch V Sindic CJ Fernández O. Effectiveness and safety of natalizumab in real-world clinical practice: review of observational studies. Clin Neurol Neurosurg. (2016) 149:55–63. 10.1016/j.clineuro.2016.07.001 27475049

[20] Kappos L Bates D Edan G Eraksoy M Garcia-Merino A Grigoriadis N. Natalizumab treatment for multiple sclerosis: updated recommendations for patient selection and monitoring. Lancet Neurol. (2011) 10:745–58. 10.1016/S1474-4422(11)70149-1 21777829

[21] Fox RJ Cree BA De Sèze J Gold R Hartung HP Jeffery D. MS disease activity in RESTORE: a randomized 24-week natalizumab treatment interruption study. Neurology. (2014) 82:1491–8. 10.1212/WNL.0000000000000355 24682966 PMC4011468

[22] Cohan SL Moses H Calkwood J Tornatore C LaGanke C Smoot KE. Clinical outcomes in patients with relapsing-remitting multiple sclerosis who switch from natalizumab to delayed-release dimethyl fumarate: a multicenter retrospective observational study (STRATEGY). Mult Scler Relat Disord. (2018) 22:27–34. 10.1016/j.msard.2018.02.028 29524759

[23] Zurawski J Flinn A Sklover L Sloane JA. Relapse frequency in transitioning from natalizumab to dimethyl fumarate: assessment of risk factors. J Neurol. (2016) 263:1511–7. 10.1007/s00415-016-8162-8 27193310

[24] Cohan SL Edwards K Lucas L Gervasi-Follmar T O'Connor J Siuta J. Reducing return of disease activity in patients with relapsing multiple sclerosis transitioned from natalizumab to teriflunomide: 12-month interim results of teriflunomide therapy. Mult Scler J Exp Transl Clin. (2019) 5:2055217318824618. 10.1177/2055217318824618 30729028 PMC6350141

[25] Guger M Enzinger C Leutmezer F Kraus J Kalcher S Kvas E. Switching from natalizumab to fingolimod treatment in multiple sclerosis: real life data from the austrian ms treatment registry. J Neurol. (2019) 266:2672–7. 10.1007/s00415-019-09464-0 31312958

[26] Giovannoni G Marta M Davis A Turner B Gnanapavan S Schmierer K. Switching patients at high risk of pml from natalizumab to another disease-modifying therapy. Pract Neurol. (2016) 16:389–93. 10.1136/practneurol-2015-001355 27114560

[27] Leurs CE van Kempen ZL Dekker I Balk LJ Wattjes MP Rispens T. Switching natalizumab to fingolimod within 6 weeks reduces recurrence of disease activity in MS patients. Mult Scler. (2018) 24:1453–60. 10.1177/1352458517726381 28823223 PMC6174622

[28] Zhovtis Ryerson L Frohman TC Foley J Kister I Weinstock-Guttman B Tornatore C. Extended interval dosing of natalizumab in multiple sclerosis. J Neurol, Neurosurg Psychiatr. (2016) 87:885–9. 10.1136/jnnp-2015-312940 26917698

[29] Ryerson LZ Foley J Chang I Kister I Cutter G Metzger RR. Risk of natalizumab-associated pml in patients with ms is reduced with extended interval dosing. Neurology. (2019) 93:e1452–62. 10.1212/WNL.0000000000008243 31515290 PMC7010325

[30] Harding K Williams O Willis M Hrastelj J Rimmer A Joseph F. Clinical Outcomes of Escalation vs. Early Intensive Disease-Modifying Therapy in Patients With Multiple Sclerosis. JAMA Neurol. (2019) 76:536–41. 10.1001/jamaneurol.2018.4905 30776055 PMC6515582

[31] He A Merkel B Brown JWL Zhovits Ryerson L Kister I Malpas CB. Timing of high-efficacy therapy for multiple sclerosis: a retrospective observational cohort study. Lancet Neurol. (2020) 19:307–16. 10.1016/S1474-4422(20)30067-3 32199096

[32] Brown JWL Coles A Horakova D Havrdova E Izquierdo G Prat A. Association of initial disease-modifying therapy with later conversion to secondary progressive multiple sclerosis. JAMA. (2019) 321:175–87. 10.1001/jama.2018.20588 30644981 PMC6439772

[33] Fernández Ó. Is there a change of paradigm towards more effective treatment early in the course of apparent high-risk MS? Mult Scler Relat Disord. (2017) 17:75–83. 10.1016/j.msard.2017.07.003 29055479

[34] Sgarlata E Chisari CG D'Amico E Millefiorini E Patti F. Changes in anti-JCV antibody status in a large population of multiple sclerosis patients treated with natalizumab. CNS Drugs. (2020) 34:535–43. 10.1007/s40263-020-00716-6 32221861

